# Circulating Monocyte and Lymphocyte Populations in Healthy First-Degree Relatives of Type 2 Diabetic Patients at Fasting and during Short-Term Hyperinsulinemia

**DOI:** 10.1155/2019/1491083

**Published:** 2019-03-11

**Authors:** Michaela Šiklová, Eva Krauzová, Barbora Svobodová, Jana Kračmerová, Marek Štěpán, Michal Koc, Vladimír Štich, Lenka Rossmeislová

**Affiliations:** ^1^Department of Pathophysiology, Third Faculty of Medicine, Charles University, Prague 100 00, Czech Republic; ^2^Franco-Czech Laboratory for Clinical Research on Obesity, Third Faculty of Medicine, Prague, Czech Republic; ^3^2nd Internal Medicine Department, University Hospital Kralovske Vinohrady, Prague 100 00, Czech Republic

## Abstract

**Aim:**

The development of type 2 diabetes (T2DM) is associated with disturbances of immune status that may be reflected by alterations of the profile of circulating immune cells. In order to study whether there exists genetic predisposition to these alterations, we investigated the relative content of circulating monocyte and lymphocyte subpopulations at fasting condition and upon stimulation by short-term hyperinsulinemia in nondiabetic first-degree relatives (FDR) of T2DM patients and in control subjects.

**Materials and Methods:**

19 nondiabetic (FDR) and 19 control subjects without a family history of diabetes (all men) matched for age and BMI underwent 2-hour hyperinsulinemic-euglycemic clamp. Blood samples taken before and at the end of the clamp were used for the flow cytometry analysis of lymphocyte and monocyte populations and for the assessment of cytokine levels.

**Results:**

At fasting conditions, FDR showed a higher CD4/CD8 ratio of peripheral lymphocytes, a higher percentage of Th17 lymphocytes, and a lower content of intermediate monocytes when compared to controls. The CD4/CD8 ratio correlated with fat mass, insulin, and HOMA-IR in the entire group of subjects. Hyperinsulinemia decreased a relative content of peripheral CD4+ and increased a relative content of CD8+ T lymphocytes, thus decreasing the CD4/CD8 ratio by 18-22% in both groups of subjects. In FDR but not in controls, the decrease of CD4+ T lymphocyte content was partially based on the decrease of T_H_2 and T_H_17 lymphocyte subpopulations. In control subjects but not in FDR, the number of intermediate monocytes has declined in response to hyperinsulinemia.

**Conclusion:**

The alterations of the CD4/CD8 lymphocyte ratio, relative content of T_H_17 cells, and intermediate monocytes in FDR are features of genetic predisposition to T2DM and may play a role in pathogenesis of T2DM. Short-term hyperinsulinemia affected mostly the immune cell populations deregulated in FDR subjects, which suggests important interplay between immune system homeostasis and insulin levels.

## 1. Introduction

Type 2 diabetes (T2DM) and its complications are associated with a systemic low-grade inflammation manifested by higher systemic levels of proinflammatory cytokines, such as IL-6, IL-1*β*, or TNF*α* [1]. The increased levels of cytokines can originate from metabolically challenged tissues such as liver, pancreas, and adipose tissues or from circulating immune cells, whose phenotype and numbers (or relative content) are sensitive to metabolic challenge. Higher fasting glucose concentration and occurrence of T2DM were positively associated with higher abundance of memory CD4+ T-lymphocytes [2] and T_H_1 and T_H_17 lymphocytes in the blood (reviewed in [3]). Furthermore, a recent meta-analysis showed that T2DM patients have decreased the content of peripheral CD4/CD25/Foxp3 regulatory T-cells and concomitantly decreased circulating levels of anti-inflammatory cytokine IL-10 [4]. Thus, the dysregulation of immune response might be associated and contribute to T2DM development.

It was suggested previously that the primary trigger of immune cell activation and thus low grade inflammation could be both acute and prolonged periods of hyperglycemia in obese patients and other patients at risk of T2DM development [5, 6]. Insulin, a hormone released in response to increased glucose levels, is one of the factors, which may alleviate the detrimental effects of hyperglycemias on immune cell phenotypes. Indeed, insulin was shown to inhibit the NF*κ*B pathway [7] or downregulate circulating levels of acute-phase proteins [8], and thus, it is believed to have anti-inflammatory effects. In *in vitro* treatment of peripheral blood mononuclear cells (PBMC) with insulin, it induced a differentiation of lymphocytes to T-helper type 2 (T_H_2) phenotype and decreased the ratio of T_H_1 to T_H_2 cells [9]. In response to acute hyperinsulinemia, a decrease of TNF*α*, IL-8, and IL-18 circulating levels was observed in healthy lean subjects, while in first-degree relatives (FDR) of type 2 diabetic patients, this anti-inflammatory response was blunted [10]. Thus, it seems likely that the dysregulation of the immune response to insulin/nutritional stimuli represents one part of the genetic predisposition to T2DM.

To test this hypothesis, we aimed to investigate the changes in lymphocyte and monocyte populations in association with genetic predisposition to T2DM and the response of these cells to short-term hyperinsulinemia in healthy FDR of T2DM when compared to control subjects.

## 2. Material and Methods

### 2.1. Subjects, Dietary Protocol, and Clinical Examination

38 lean men participated in the study. Two groups of subjects matched for age and BMI were recruited: (1) nondiabetic first-degree relatives of T2DM patients (FDR; *n* = 19)—family history of diabetes was considered as follows: two first-degree relatives (parents, siblings) or one first-degree and one or more second-degree relatives (grandparents, uncle, aunt) were diagnosed with T2DM; and (2) control group—subjects without any family history of diabetes (CON; *n* = 19).

All subjects were generally healthy and nonobese and did not use any prescription drugs, as determined by medical history and laboratory findings. The exclusion criteria for both groups were weight change more than 3 kg within 3 months preceding the study, smoking, hypertension, diabetes, hyperlipidemia, illicit drug, or alcohol abuse. Subjects were examined at a certified laboratory starting at 8 am after an overnight fast. Body weight and waist and hip circumferences were measured, and body composition was assessed by bioimpedance (QuadScan 4000, Bodystat, Douglas, British Isles). The study was approved by the Ethical Committee of the Third Faculty of Medicine, Charles University, in Prague, and all subjects gave their informed consent before the start of the study.

### 2.2. Euglycemic-Hyperinsulinemic Clamp

The euglycemic-hyperinsulinemic clamp was performed according to the de Fronzo method [11]. A catheter for insulin and glucose infusion was inserted into an antecubital vein, and the second catheter for blood sampling was placed in the same location of the ipsilateral arm. The forearm was kept wrapped in a heated blanket to provide arterialization of venous blood. Priming dose plus continuous infusion of crystalline human insulin (Actrapid Human, Novo, A/S, Bagsvaerd, Denmark), 40 mU/m^2^ body area/min, was given for 120 min. Euglycemia (at the level of the individual fasting blood glucose concentration) was maintained by a variable 20% glucose infusion. The infusion rate was adjusted according to arterialized plasma glucose levels measured every 5 minutes (Beckman Glucose analyzer, Beckman Instruments, Fullerton, CA, USA).

### 2.3. Flow Cytometry Analysis of Immune Cell Populations

The subpopulation of blood cells representing lymphocytes and monocytes was analyzed according to their size and granularity. To detect specific surface antigens, the whole blood samples were stained with fluorescence-labelled monoclonal antibodies (FITC-conjugated antibody CD4, CD14; PE-conjugated antibody CD3, and CD163; PerCP-conjugated antibody CD45; APC-conjugated antibody CD8, CD36, TLR2, and CD196; APC-Cy7-conjugated antibody CD16; PE-Cy7-conjugated antibody CCR2 and CD25; BV421-conjugated antibody CD183; and CD194; all except CD163 from BD Bioscience, US; CD163 from Exbio, CZ) for 30 min at room temperature. After staining, erythrocytes were lysed by erythrocyte lysis buffer for 15 min at room temperature. The cells were then washed with PBS and analyzed on a FACSVerse flow cytometer and by BD FACSuite Software 1.0.6 (BD Biosciences). The number of immune cells in the analyzed populations was expressed as the percentage of gated events related to CD45, CD45+/14+ (monogate), or CD3+ (lymphogate) events. Background was set up to 5% of positive cells of isotype control.

T cells were distinguished by standard FSC/SSC position “lymphogate” and by positivity for pan T cell marker CD3. CD4-positive T cells were considered T helper (T_H_) cells and CD8-positive cells as T cytotoxic (Tc). T_H_1 cells were defined as CD4+/194-/196-/183+ (positive for CXCR3), T_H_2 were defined as CD4+/194+/196-/183- (positive for CCR4), and T_H_17 were defined as CD4+/194+/196+/183- (positive for CCR4/CCR6). Regulatory T cells (Tregs) were identified as CD4/CD25high/CD127low cells. Monocytes were distinguished by standard FSC/SSC position and by positivity for CD45, CD14, and CD16 markers.

### 2.4. Analysis of Plasma

Plasma concentrations of glucose, insulin, lipids, and nonesterified fatty acids (NEFA) were determined using standard biochemical methods. Cytokines in the plasma were analyzed using xMAP technology (High Sensitivity Human T-cell Kit: IL-4, IL-6, IL-10, IL-12, IL-17A, and TNF*α*; Merck-Millipore, USA) on the MagPIX instrument or using ELISA (Duoset MCP1, R&D Systems, Minneapolis, USA).

### 2.5. Statistical Analysis

Data are presented as the means ± SEM. Statistical analysis was performed using GraphPad Prism 7.0 for Windows (La Jolla, USA). Differences between the baseline values and between the responses to hyperinsulinemia in the two groups of subjects, the FDR and control group, were analyzed by two-way ANOVA, with Bonferroni post hoc analysis. Correlations at the baseline were expressed as Pearson's correlation coefficient. The level of significance was set at *p* < 0.05.

## 3. Results

### 3.1. Baseline Characteristics of the Subjects and Immune Cell Populations

The anthropometric and biochemical parameters of the subjects are listed in Table 1. The groups were not different in age, BMI, fat mass, waist circumference, and lipid parameters. FDR had a higher baseline glucose, fasting insulin, and insulin resistance as evaluated by the HOMA-IR index.

**Figure 1:**
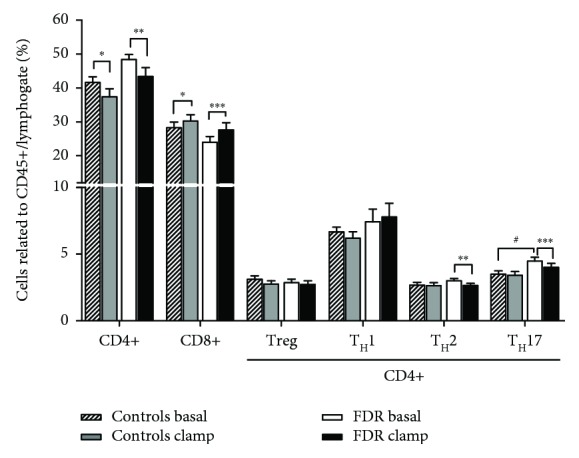
Effect of hyperinsulinemia on lymphocyte populations in the blood in FDR and in control subjects. Lymphocyte populations are expressed as the percentage of positive cells related to CD45/lymphogate. ^∗∗∗^*p* < 0.001. ^∗∗^*p* < 0.01. ^∗^*p* < 0.05, significant change during the euglycemic-hyperinsulinemic clamp. ^$^*p* < 0.05, significant difference between FDR and in control subjects at baseline. Striped bars: baseline preclamp levels in controls. Grey bars: postclamp levels at the end (2 hours) of the clamp in controls. White bars: baseline preclamp levels in FDR. Black bars: postclamp levels at the end (2 hours) of the clamp in FDR. FDR: first-degree relatives.

At the fasting state (baseline), the T lymphocyte populations in the blood were not different between the groups, except for T_H_17 lymphocytes, which were higher in FDR (Figure 1). Moreover, the ratio of CD4/CD8 T lymphocytes was higher in the FDR group when compared to controls (2.29 ± 0.24 vs. 1.61 ± 0.14, *p* = 0.04, respectively). The relative content of CD4+ T cells and CD4/CD8 ratio correlated positively with FM in the whole group of subjects (FDR + controls) (Table 2). The CD4/CD8 ratio correlated positively to insulin and HOMA-IR and negatively to IL-4 cytokine (Table 2).

**Figure 2:**
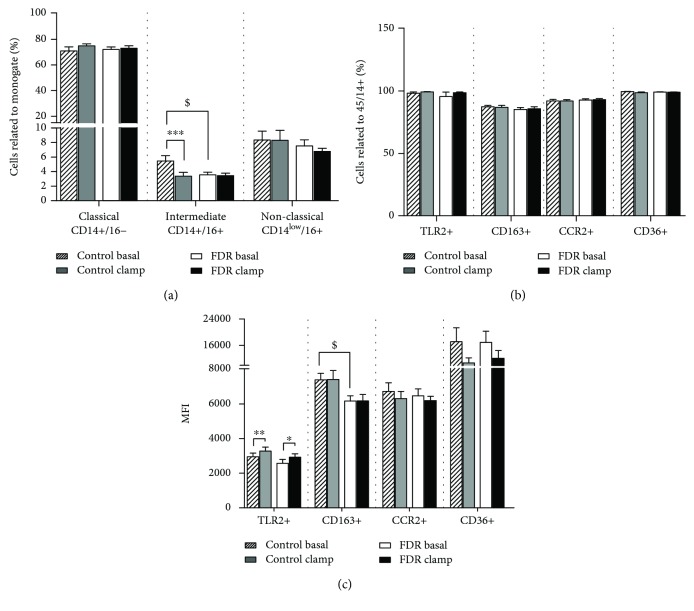
Effect of hyperinsulinemia on monocyte populations in the blood in FDR and in control subjects: (a) classical, intermediate, and nonclassical monocytes expressed as the percentage of positive cells related to CD45/14+; (b) monocyte populations expressing markers TLR2, CD163, CCR2, and CD36 expressed as the percentage of positive cells related to CD45/14+; (c) expression of markers TLR2, CD163, CCR2, and CD36 on monocyte subsets expressed as MFI ^∗∗^*p* < 0.01; ^∗^*p* < 0.05, significant change during the euglycemic-hyperinsulinemic clamp; ^$^*p* < 0.05, significant difference between FDR and control subjects at baseline; striped bars—baseline preclamp levels in controls; grey bars—postclamp levels at the end (2 hours) of the clamp in controls; white bars—baseline preclamp levels in FDR; black bars—postclamp levels at the end (2 hours) of the clamp in FDR. FDR: first-degree relatives; MFI: mean fluorescence intensity.

The monocyte populations in the blood did not differ between the groups at fasting conditions (Figures 2(a) and 2(b)), except for intermediate monocytes (CD45/14+/16+), which were lower in FDR compared to controls (Figure 2(a)). Moreover, in FDR, expression levels of CD163 (MFI) in CD163+ monocytes were lower compared to control subjects (Figure 2(c)). The relative content of the CD163+ monocytes was positively correlated to insulin sensitivity as evaluated by hyperinsulinemic-euglycemic clamp (M_FFM_), while the relative content of classical monocytes correlated to fasting insulin levels and HOMA-IR in the whole group of subjects (Table 3).

### 3.2. Effect of Hyperinsulinemia on Circulating Lymphocytes

In response to hyperinsulinemia, a decrease in the relative content of CD4+ T helper cells and an increase of relative content of CD8+ cytotoxic T cells were detected in both groups of subjects. Thus, the CD4/CD8 ratio decreased from 2.29 ± 0.24 to 1.90 ± 0.24 (*p* < 0.0001) in FDR and from 1.61 ± 0.14 to 1.37 ± 0.16 (*p* = 0.004) in the control group, with no significant difference in this response between the groups. The relative content of T_H_2 and T_H_17 lymphocytes dropped in FDR (Figure 1), while no change in these T_H_-cell subpopulations was observed in control subjects (Figure 1).

### 3.3. Effect of Hyperinsulinemia on Circulating Monocytes

No change in the relative content of classical (CD45/14+/16-) and nonclassical monocytes (CD45/14^low^/16+) was observed in response to hyperinsulinemia in either group. Intermediate monocyte (CD45/14+/16+) count decreased after 2 h of sustained hyperinsulinemia in the control group only (Figure 2(a)). No changes in monocyte subpopulations expressing TLR2, CD163, CCR2, and CD36 were observed (Figure 2(b)).

An increased of expression (expressed as MFI) of the TLR2 scavenger receptor was detected on monocytes in response to hyperinsulinemia in both groups of subjects (Figure 2(c)).

### 3.4. Plasma Cytokines

No difference in the plasma levels of IL-4, IL-6, IL-10, IL-12, IL-17, TNF*α*, and MCP-1 was observed in baseline and in response to hyperinsulinemia in either group of subjects (Table 3). Baseline IL-4 concentrations correlated positively with the relative content of intermediate monocytes and negatively with CD4+ cells and with the CD4/CD8 ratio when analyzed in all subjects (Table 2).

## 4. Discussion

In this study, we investigated the relative content and polarization of circulating monocyte and T lymphocyte populations and the effect of hyperinsulinemia on these immune cells, in respect to genetic predisposition to T2DM.

Compared to control subjects without genetic predisposition to T2DM, FDR entering our study exhibited mildly higher glucose and insulin fasting levels (and concomitantly higher HOMA-IR index) representing early risk factors for the development of T2DM. These findings were in agreement with the previously described difference between nonobese FDR and control subjects [12–14]. Notably, insulin and HOMA-IR levels correlated positively to the CD4/CD8 lymphocyte ratio. Previously, the CD4/CD8 ratio was found to be increased in the new-onset streptozocin-treated diabetic mice [15] and also in T1DM patients and their first-degree relatives [16]. Moreover, in humans, the genetic predisposition to T1DM was associated with genetic variation at MHC locus, which seems to be responsible for peripheral blood CD4+/CD8+ T lymphocyte homeostasis [17]. In T1DM patients, the *β*-cell destruction is caused by the autoimmune attack of CD8+ activated by cytokines released by CD4/T_H_1 cells and macrophages [18]. Interestingly, the increased infiltration of immune cells together with increased expression of proinflammatory cytokines, such as IL-1*β*, TNF*α*, was found also in islets in T2DM subjects [19, 20]. Thus, it was suggested that the development of the local proinflammatory state is an important pathophysiological mechanism in autoimmune-induced apoptosis of pancreatic *β*-cells not only in T1DM [18] but also in T2DM subjects as well [21, 22]. The findings observed in our study with FDR of T2DM subjects and other studies with T1DM patients and their FDR may suggest that the imbalance in the CD4/CD8 lymphocyte ratio might be figured among factors associated with diabetes development.

Moreover, the higher percentage of baseline Th17 lymphocytes found in the FDR group fits to the concept of the proinflammatory state preceding/contributing to diabetes onset, since T_H_17 T-cells producing IL-17 were associated with proinflammatory reactions and diabetic complications [23] and increased levels of T_H_1 and T_H_17 circulating T-cells were reported also in T2DM subjects [4, 24, 25].

On the other hand, there was no difference between the two groups in relative distribution of classical and nonclassical monocyte populations, while the “intermediate” CD14+/16+ monocytes were lower in the FDR group. Intermediate monocytes express more than 80% of genes and surface markers at levels between classical and nonclassical monocytes [26]. They are considered proinflammatory and were associated with several inflammatory diseases [27, 28]. However, their role in metabolic diseases is not fully understood. In several studies with obese subjects, no difference or increased levels of peripheral intermediate monocytes were reported [29, 30]. On the other hand, in women with gestational diabetes, intermediate monocytes were shown to be lower when compared to healthy controls [31]. This monocyte subpopulation was shown to produce the anti-inflammatory cytokine IL-10, and thus, the function of this monocyte population might be considered immunomodulatory [31]. In agreement with this view, the content of intermediate monocytes correlated positively with anti-inflammatory cytokine IL-4 in our study. Nevertheless, the role of intermediate monocytes in diabetes development should be warranted in future studies.

In order to investigate further regulation of circulating lymphocytes and monocytes, we investigated responses of these immune cell populations to insulin action; namely, we studied these populations during short-term hyperinsulinemia induced by hyperinsulinemic-euglycemic clamp. The decrease of the CD4/CD8 ratio of T-lymphocytes evoked by short-term hyperinsulinemia in both groups of subjects suggests a marked role of insulin in the balance of these two cell populations. In fact, the insulin-stimulated decrease of CD4+ T-cells was accompanied by a decline of the relative content of T_H_2 and T_H_17 subpopulations in the FDR group to the levels seen in the control group. This could suggest that in the absence of elevated glucose, hyperinsulinemia is able to normalize the balance between T_H_ subsets, while under more physiological or chronic conditions, this protective role of insulin may be masked or overridden by the proinflammatory effects of glucose and other metabolites/factors. On the other hand, hyperinsulinemia drove the decline of intermediate monocytes in control subjects to the level seen in FDR. Although the function of intermediate monocytes in metabolism is questionable as mentioned above, this result could suggest that both acute and chronic hyperinsulinemia is an important regulator of this monocyte subtype.

Further, an increase of expression (MFI) of TLR2, a well-characterized immune scavenger receptor, on monocytes was observed in response to hyperinsulinemia. TLR2 was shown to trigger low-grade chronic inflammation and activation of macrophages, present in obesity, T2DM, or atherosclerosis [32], and a higher TLR2 expression was shown in monocytes of T2DM patients compared with control subjects [33]. Thus, insulin exerts complex regulation of monocytes and T lymphocytes, which warrants further studies in this issue.

Finally, our study did not show any effect of the genetic predisposition on fasting plasmatic levels of various cytokines similarly as described previously [10, 34]. This suggests that low-grade inflammation on the systemic level is not induced before the T2DM development in FDR. However, contrary to our results, the study of Ruotsalainen et al. [10] demonstrated the differential response of cytokines to hyperinsulinemia in controls and FDR (i.e., decrease of IL10, TNF*α*, IL8, and IL18 levels selectively in control subjects). The lack of the acute effect of hyperinsulinemia on circulating levels of analyzed cytokines in our study could imply the gender effect on this response, as we investigated only men, while the study of Ruotsalainen et al. [10] included both genders.

## 5. Conclusions

In conclusion, we demonstrated a shift in the peripheral CD4/CD8 lymphocytes ratio, T_H_17 cells, and intermediate monocytes in subjects genetically predisposed to T2DM in comparison to controls. The imbalance in these immune cell populations might be features of genetic predisposition to T2DM and may play a role in pathogenesis of T2DM. Interestingly, short-term hyperinsulinemia affected mostly the immune cell populations deregulated in FDR subjects, which suggests an important interplay between immune system homeostasis and insulin levels.

## Figures and Tables

**Table 1:** The anthropometrical and biochemical parameters of the 2 groups of subjects.

	Controls (*n* = 19)	FDR (*n* = 19)	*p* value
Age (years)	36 ± 1.1	37 ± 1.3	0.469
Weight (kg)	81.9 ± 2.2	83.6 ± 1.1	0.515
BMI (kg/m^2^)	24.7 ± 0.5	25.2 ± 0.4	0.401
Waist (cm)	84.2 ± 1.3	86.0 ± 1.3	0.341
Fat mass (%)	16.6 ± 0.9	18.6 ± 0.9	0.117
Fat-free mass (kg)	68.1 ± 1.6	67.9 ± 0.8	0.948
HDL (mmol/l)	1.36 ± 0.06	1.26 ± 0.07	0.313
TG (mmol/l)	1.06 ± 0.23	1.15 ± 0.27	0.808
Total cholesterol (mmol/l)	4.3 ± 0.3	4.7 ± 0.2	0.209
Ureic acid (*μ*mol/l)	346 ± 16	334 ± 12	0.725
Glucose (mmol/l)	5.2 ± 0.1	5.6 ± 0.1	0.019
Insulin (mU/l)	5.2 ± 0.6	8.4 ± 1.1	0.018
HOMA-IR	1.2 ± 0.2	2.1 ± 0.3	0.015
M_FFM_ (mg/kgFFM/min)	8.1 ± 0.5	7.4 ± 0.7	0.461

Data are presented as the mean ± SEM. Statistical difference between the groups evaluated by a nonpaired *t*-test, *p* < 0.05. BMI: body mass index; HDL: high-density cholesterol; TG: triglycerides; HOMA-IR: homeostasis model assessment of insulin resistance; M_FFM_: glucose disposal related to fat-free mas.

**Table 2:** Correlations between clinical parameters and circulating immune cells at baseline in all subjects.

Clinical parameter	Immune population	*p* value	Correl. coef.
FM	CD45/3/4+	0.016	0.399
	CD4/CD8 ratio	0.020	0.387

Insulin	Classical monocytes	0.026	0.376
	CD4/CD8 ratio	0.048	0.387

HOMA-IR	Classical monocytes	0.029	0.369
	CD4/CD8 ratio	0.035	0.348

M_FFM_	CD45/14/163+	0.045	0.341

IL-4	CD45/3/4+	0.014	-0.422
	CD4/CD8 ratio	0.010	-0.451
	Intermediate monocytes	0.020	0.418

Data are presented as Pearson's correlation coefficient and *p* value. FM: fat mass; HOMA-IR: homeostasis model assessment of insulin resistance; M_FFM_: glucose disposal related to fat-free mas; IL-4: interleukin 4.

**Table 3:** The concentrations of cytokines in the plasma before and after the hyperinsulinemic clamp in the 2 groups of subjects.

	Controls (*n* = 18)		FDR (*n* = 18)	
	Basal	Clamp	Basal	Clamp
IL-4 (pg/ml)	20.1 ± 2.3	21.4 ± 2.1	17.4 ± 1.5	18.0 ± 1.4
IL-6 (pg/ml)	0.99 ± 0.15	1.19 ± 0.16	0.89 ± 0.10	0.96 ± 0.14
IL-10 (pg/ml)	4.2 ± 0.6	4.6 ± 0.8	3.9 ± 0.7	3.4 ± 0.7
IL-12 (pg/ml)	1.6 ± 0.2	1.8 ± 0.2	1.7 ± 0.2	1.7 ± 0.2
IL-17 (pg/ml)	6.6 ± 0.7	6.9 ± 0.8	5.4 ± 0.6	5.5 ± 0.6
TNF*α* (pg/ml)	1.7 ± 0.1	1.9 ± 0.2	1.6 ± 0.2	1.5 ± 0.2
MCP-1 (pg/ml)	42.6 ± 5.8	34.8 ± 5.6	36.7 ± 5.0	30.8 ± 5.1

Data are presented as the mean ± SEM. IL: interleukin; TNF*α*: tumor necrosis factor alpha; MCP1: monocyte chemotactic protein 1.

## Data Availability

The (lymphocyte and monocyte populations measured by flow cytometry and plasma cytokine levels) data used to support the findings of this study are included within the article. The original (raw) data of this study are available from the corresponding author upon request.
